# Assessment of Filtration Bleb and Endplate Positioning Using Magnetic Resonance Imaging in Eyes Implanted with Long-Tube Glaucoma Drainage Devices

**DOI:** 10.1371/journal.pone.0144595

**Published:** 2015-12-07

**Authors:** Ichiya Sano, Masaki Tanito, Koji Uchida, Takashi Katsube, Hajime Kitagaki, Akihiro Ohira

**Affiliations:** 1 Division of Ophthalmology, Matsue Red Cross Hospital, Matsue, Japan; 2 Department of Ophthalmology, Shimane University Faculty of Medicine, Izumo, Japan; 3 Department of Radiology, Shimane University Faculty of Medicine, Izumo, Japan; Bascom Palmer Eye Institute, University of Miami School of Medicine;, UNITED STATES

## Abstract

**Background:**

To evaluate ocular fluid filtration and endplate positioning in glaucomatous eyes with long-tube glaucoma drainage devices (GDDs) using magnetic resonance imaging (MRI) and the effects of various factors on postoperative intraocular pressure (IOP).

**Methods:**

This observational case series included 27 consecutive glaucomatous eyes (18 men, 7 women; mean age ± standard error, 63.0±2.0 years) who underwent GDD implantation (n = 8 Ahmed Glaucoma Valves [AGV] and n = 19 Baerveldt Glaucoma Implants [BGI]). Tubes were inserted into the pars plana in 23 eyes and anterior chamber in 4 eyes. Six months postoperatively, high-resolution orbital images were obtained using 3-Tesla MRI with head-array coils, and the filtering bleb volume, bleb height, and distances between the anterior endplate edge and corneal center or limbus or between the endplate and orbital wall were measured.

**Results:**

In MR images obtained by three-dimensional fast imaging employing steady-state acquisition (3D-FIESTA) sequences, the shunt endplate was identified as low-intensity signal, and the filtering bleb was identified as high-intensity signals above and below the endplate in all eyes. The 6-month-postoperative IOP level was correlated negatively with bleb volume (r = -0.4510, *P* = 0.0182) and bleb height (r = -0.3954, *P* = 0.0412). The postoperative IOP was significantly (*P* = 0.0026) lower in BGI-implanted eyes (12.2±0.7 mmHg) than AGV-implanted eyes (16.7±1.2 mmHg); bleb volume was significantly (*P* = 0.0093) larger in BGI-implanted eyes (478.8±84.2 mm^3^) than AGV-implanted eyes (161.1±52.3 mm^3^). Other parameters did not differ.

**Conclusions:**

The presence of intraorbital/periocular accumulation of ocular fluid affects postoperative IOP levels in eyes implanted with long-tube GDDs. Larger filtering blebs after BGI than AGI implantations explain lower postoperative IOP levels achieved with BGI than AGV. The findings will contribute to better understanding of IOP reducing mechanism of long-tube GDDs.

## Introduction

Glaucoma is a chronic disease characterized by progressive loss of the retinal nerve fiber layer and associated visual field loss [1,2], and the intraocular pressure (IOP) is currently the only modifiable risk factor [3]. The treatment approach to decrease the IOP traditionally starts with topical medications followed by laser surgery and eventually invasive surgery in patients refractory to earlier interventions. Trabeculectomy remains the gold standard when non-invasive techniques have failed, although newer techniques are currently in use and under investigation [4,5].

Recently, implantation of glaucoma drainage devices (GDDs) made of different materials are being used increasingly to treat early and advanced glaucoma worldwide [6,7] and more recently in Japan [8–10]. Among the various types of GDDs, the device with an endplate and long tube has been successfully used in complicated glaucoma cases, including neovascular glaucoma, aphakic and pseudophakic glaucoma, postpenetrating keratoplasty glaucoma, pediatric glaucoma, and uveitic glaucoma [11–14]. The long-tube types of GDDs currently used often include the Ahmed Glaucoma Valve (AGV) (New World Medical, Rancho Cucamonga, CA), the Baerveldt glaucoma implant (BGI) (Abbott Medical Optics, Abbott Park, IL), and the Molteno Implant (Molteno Ophthalmic, Dunedin, New Zealand) [6]. The aim of long-tube GDDs surgery is to create a functioning bleb around the endplate portion of the implant at the bulbar equator to facilitate flow of aqueous humor through the bleb wall. Compared to the bleb after trabeculectomy, because of its posterior formation, the bleb formed after long-tube GDD surgery is difficult to assess during regular ophthalmic examinations such as slit-lamp, anterior-segment optical coherence tomography (OCT), or ultrasound biomicroscopy (UBM).

The purpose of the current study was to evaluate the intraorbital status of ocular fluid filtration and endplate positioning in glaucomatous eyes implanted with either the AGV or BGI, two of the most common types of long-tube GDDs, using magnetic resonance imaging (MRI). MRI seems to have advantages for assessing bleb formation around the equator of eyeball with a large field of view without depth limitation, although there are some challenges like limited spatial resolution. The possible effects of various MRI-measured parameters including bleb volume, bleb height, and plate positioning on the postoperative IOP also were assessed.

## Subjects and Methods

### Subjects

This observational case series included 27 consecutive refractory glaucomatous eyes of 25 Japanese subjects (mean age ± standard error of the mean (SEM), 63.0±2.0 years; 18 men, 7 women) who underwent long-tube GDD implantation surgeries (8 AGVs and 19 BGIs) at Shimane University Hospital between December 2009 and February 2013 to control IOP (Table 1). The patients underwent orbital MRI examination to assess the bleb status 6 months postoperatively. The study adhered to the tenets of the Declaration of Helsinki and the institutional review boards of Shimane University Hospital reviewed and approved the research. Preoperatively, all subjects provided written informed consent for use of clinical data regarding the glaucoma treatment obtained during the follow-up periods.

**Table 1:** Demographic Subject Data.

Case	Age (years)	Sex	Eye	Glaucoma Type	Shunt Type	Plate Size (mm^2^)	Endplate Position	Tube Insertion	Preop IOP (mmHg)	Postop IOP (mmHg)	IOP Reduction (mmHg)	Preop Medication Score	Postop Medication Score
1	70	M	L	Other	AGV	184	ST	PP	30	15	15	3	2
2	51	M	L	Other	AGV	184	IT	PP	31	21	10	3	2
3	60	M	L	NVG	AGV	184	IT	PP	42	15	27	1	2
4	56	M	R	POAG	AGV	184	IT	PP	43	21	22	4	3
5	57	M	R	POAG	AGV	184	IN	PP	34	19	15	3	3
6	50	M	L	NVG	AGV	184	IT	AC	51	13	38	3	1
7	56	M	R	NVG	AGV	184	IT	PP	30	13	17	3	3
8	76	F	R	POAG	AGV	184	IT	PP	35	17	18	4	0
9	77	F	R	Other	BGI	350	ST	PP	30	11	19	3	0
10	63	M	L	NVG	BGI	350	IT	PP	32	17	15	3	3
11	77	F	R	Other	BGI	350	IT	PP	37	9	28	3	0
12	63	F	R	POAG	BGI	350	IT	PP	29	8	21	3	3
13	64	F	L	POAG	BGI	350	IT	PP	26	11	15	4	3
14	75	M	R	Other	BGI	350	IT	PP	33	11	22	4	0
15	69	M	R	POAG	BGI	350	IT	PP	38	11	27	3	1
16	65	M	R	Other	BGI	350	IT	PP	45	9	36	4	3
17	60	F	R	Other	BGI	350	IT	PP	63	13	50	3	2
18	51	M	R	POAG	BGI	250	ST	PP	23	20	3	3	3
19	73	M	L	Other	BGI	250	IT	PP	40	11	29	3	0
20	65	F	L	NVG	BGI	350	IT	AC	55	12	43	3	0
21	78	M	L	NVG	BGI	250	ST	AC	44	12	32	2	0
22	73	M	R	NVG	BGI	350	ST	PP	67	17	50	3	3
23	71	M	R	POAG	BGI	350	IT	PP	29	11	19	5	1
24	38	M	R	Other	BGI	350	IT	PP	50	10	40	3	3
25	59	F	L	POAG	BGI	350	IT	PP	24	13	11	3	0
26	56	M	R	Other	BGI	350	IT	PP	42	11	31	4	2
27	48	M	R	POAG	BGI	350	IT	AC	45	15	30	3	3
Mean	63.0								38.8	13.6	25.3	3.2	1.7
SEM	2.0								2.2	0.7	2.3	0.1	0.2

MRI, magnetic resonance imaging; IOP, intraocular pressure; M, male; F, female; L, left eye, R, right eye; NVG, neovascular glaucoma; POAG, primary open-angle glaucoma; AGV, Ahmed Glaucoma Valve; BGI350, Baerveldt Glaucoma Implant; ST, superotemporal; IT, inferotemporal; IN; inferonasal; PP, pars plana tube insertion; AC, anterior chamber tube insertion; Preop, preoperative; Postop, postoperative; SEM, standard error of mean.

### Surgical Technique

One surgeon (MT) performed all surgeries. The tube was inserted into the pars plana in 23 eyes and the anterior chamber in four eyes. The endplate was placed in the superotemporal quadrant in five eyes, the inferotemporal quadrant in 21 eyes, and inferonasal quadrant in one eye based on the availability of surgical space due to previous ocular surgeries. The models used were eight AGV FP-7 (plate size, 184 mm^2^), 16 BGIs 101–350 (plate size, 350 mm^2^), and three BGIs 103–250 (plate size, 350 mm^2^) (Table 1).

After the fornix-based localized conjunctival peritomy was created and standard sub-Tenon anesthesia with 2% lidocaine was administered, the endplate was inserted into the subconjunctival pocket between the two rectus muscles and fixed on the scleral surface with two 5–0 polyester sutures about 8.5 mm from the corneal limbus for the AGV and 10 mm from the corneal limbus for the BGI. Under a half-thickness rectangular scleral flap, a scleral tunnel was created with a 23-gauge needle at the surgical limbus for insertion of the anterior chamber tube and 3.5 mm posterior to the surgical limbus for pars plana insertion. The trimmed tube was inserted, and the scleral flap was secured with interrupted 10–0 nylon sutures to cover the tube. In cases implanted with the BGIs, to avoid early postoperative hypotony, a 3–0 nylon suture was placed in the tube lumen as a “stent graft,” and the tube was ligated with a 7–0 polyglactin suture; two to three tube slits were created with a suture needle to avoid postoperative IOP elevation. Before the tube was inserted into the pars plana, a complete vitrectomy with meticulous shaving of the vitreous base was performed with a 23-gauge pars plana vitrectomy system. The conjunctiva was closed with 10–0 polyglactin sutures. Typically, 1.5% levofloxacin and 0.1% betamethasone were instilled topically for 1 month postoperatively in all cases except in patients with uveitic glaucoma who used betamethasone for longer periods, and the stent graft was removed before 4 months postoperatively in cases implanted with a BGI.

### Orbital MRI

**Fig 1:**
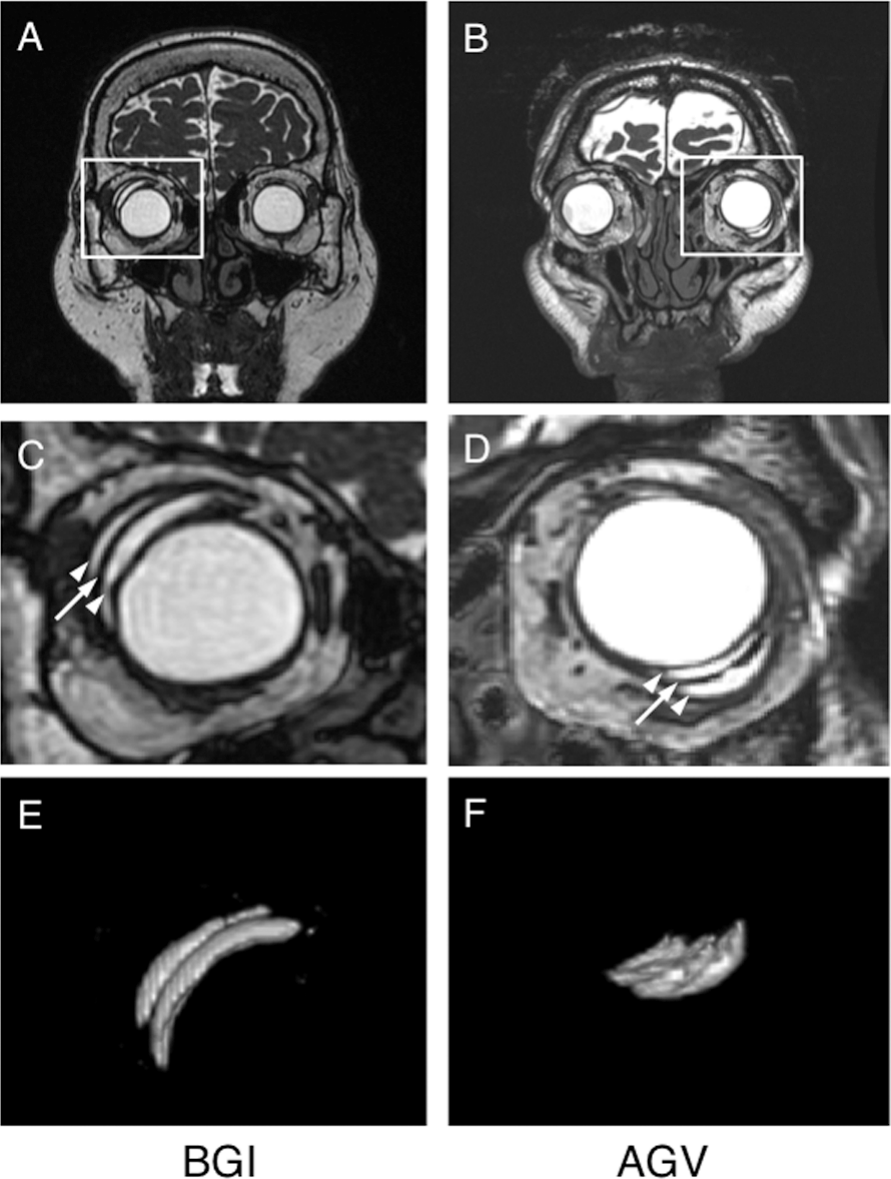
Magnetic resonance imaging (MRI) of the orbit. Coronal images show a Baerveldt Glaucoma Implant (BGI) in the superotemporal quadrant (A, C) and an Ahmed Glaucoma Valve (AGV) in the inferotemporal quadrant (B, D) 6 months postoperatively. With high magnification views (C, D), layers of fluid accumulation with the same intensity as intraocular fluid (arrowheads) are seen under (inner bleb) and over (outer bleb) the low-intensity area that corresponds to the endplate (arrows). With three-dimensional views of filtering blebs obtained by the volume-rendering process (E, F), the inner and outer blebs are seen clearly. In these cases, the bleb volume is calculated to be 646.0 mm^3^ after BGI (E) and 272.2 mm^3^ after AGV implantation (F).

High-resolution orbital images were obtained using a 3-Tesla scanner (Signa HDxt 3.0T, GE Healthcare, Milwaukee, WI) in combination with head-array coils with the sequences of three-dimensional fast imaging employing steady-state acquisition (3D-FIESTA) (Fig 1). The imaging parameters included repetition time of 5.6 ms, echo time of 2.7 ms, field-of-view of 180 mm * 180 mm, matrix size of 320 * 288, and slice thickness of 1.0 mm with the overlap thickness of 0.5 mm, resulted in in-plane resolution of 0.56 mm * 0.63 mm. Coronal plane was taken perpendicular to the plane that containing both optic nerves. The acquisition time for whole scanning was typically 3 to 3.5 min. Subjects were coached repeatedly to avoid unnecessary movements during scanning. GDDs implanted were not ferromagnetic and were MR imaging safe. The specific absorption rate of the MR imaging of this study was calculated to be less than 2.0 W/kg that indicating “less invasive” for the patients. No patient felt warmth from devices during the scans. All scans were obtained about 6 months after shunt implantation to facilitate complete healing of the conjunctiva and orbital tissues, absorption of the ligating suture, stent graft removal (with the BGI), and IOP stabilization after the hypertensive phase (with the AGV) [15].

### MRI-Measured Parameters

**Fig 2:**
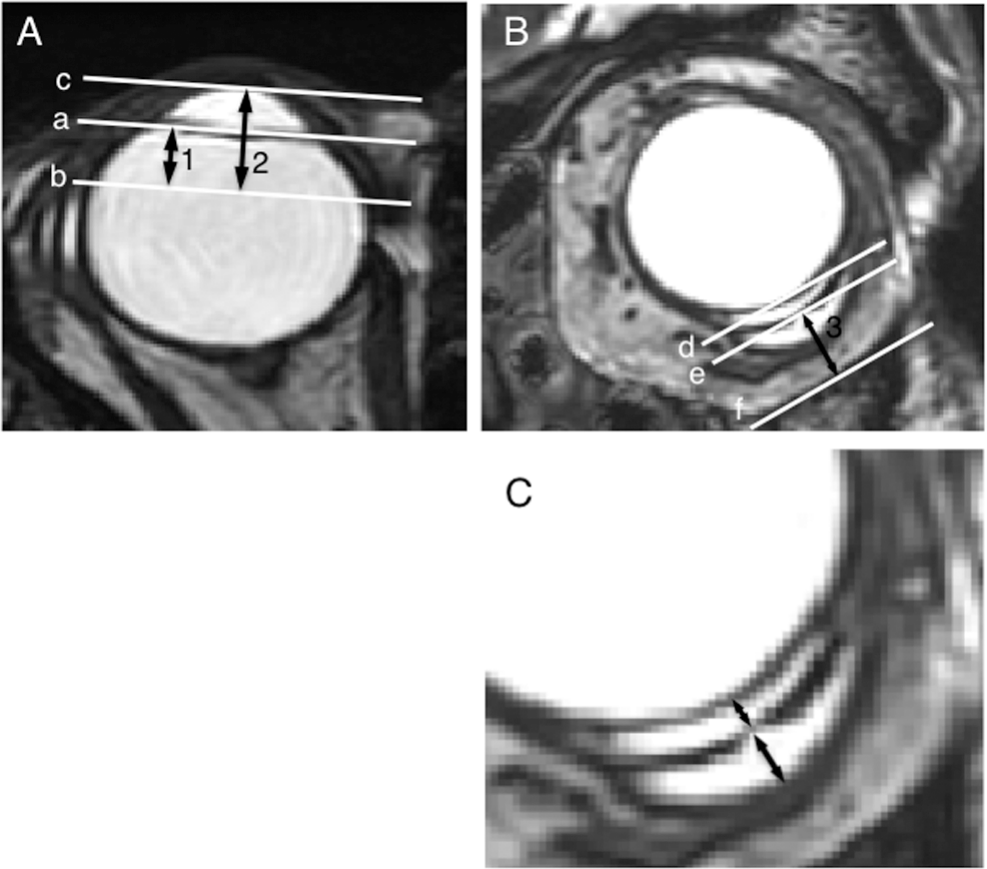
Definitions of magnetic resonance imaging-measured parameters. (A) Line a connects both edges of the corneal limbus on the axial scan image at the level of the anterior edge of the shunt is seen most anteriorly. Line b passes the anterior edge of the endplate and is parallel to line a. Line c passes the inner surface of the central cornea and is parallel to line a. The number 1 indicates the distance between lines a and b, and 2 indicates the distance between lines b and c. (B) Line d connects both edges of the endplate on the coronal scan image at the level of the width of the endplate seen is the longest. Line e passes the center of the endplate and is parallel to line d. Line f is parallel to line d and passes the nearest point from line d to the orbital bony wall. The number 3 indicates the distance between lines e and f. (C) The bleb thickness is the sum of the inner and outer bleb heights measured at the midline of the endplate on the same image used for the distance 3 measurements.

The MR images obtained were analyzed using ImageJ software (available at http://rsb.info.nih.gov/ij/; developed by Wayne Rasband, MD, PhD, National Institutes of Health, Bethesda, MD) on a personal computer. The image data saved as DICOM files were imported into the ImageJ software, the background was subtracted, and a threshold was applied to identify individual blebs in each slice. The shunt endplate was identified as a low-intensity circumlinear structure in scanning images located adjacent to the sclera in the inferotemporal, superotemporal, or inferonasal quadrant of the eyeball. The position of the endplate was evaluated by measuring three distance parameters, i.e., distance 1 indicated the distance between the corneal limbus and the anterior edge of the shunt endplate (Fig 2A), distance 2 indicated the distance between the corneal center and the anterior edge of the shunt endplate (Fig 2A), and distance 3 indicated the distance between the shunt endplate and the nearest orbital wall (Fig 2B). The bleb height also was measured and defined as the sum of the thicknesses of the inner and outer blebs observed above (orbital wall side) and beneath (scleral side) the endplate, respectively (Fig 2C). The distances were measured using the ROI manager tool. Distances 1 and 2 were measured on series of axial image that reconstructed from the coronal images, and distance 3 and bleb height were measured on series of coronal image. After choosing the positions of the filtering bleb in each slice, three-dimensional images of the bleb were constructed and the bleb volume was calculated using the volume-rendering tool Sync 3D in the Image J (Fig 1E and 1F). Based on the 3-repeated measurements in 6 subjects (3 AGV and A BGI), the coefficient of variances of each MRI-measured parameters were calculated to be 1.7% for bleb volume, 5.1% for bleb height, 4.9% for distance 1, 4.3% for distance 2, and 4.0% for distance 3.

### Statistical Analysis

The clinical parameters, including age, sex, preoperative IOP, number of antiglaucoma medications, postoperative IOP, and number of antiglaucoma medications at the time of the MRI examination were collected from the medical charts. Possible correlations between the IOP and the five MRI-measured parameters were analyzed using Spearman’s rank correlation coefficient. The clinical and MRI-measured parameters were compared between AGV and BGI groups using the Mann-Whitney U test for numerical parameters and by Fisher’s exact probability test for categorical parameters. The continuous data were expressed as the mean ± SEM. All statistical analyses were performed using the JMP version 10.02 statistical software (SAS Institute, Inc., Cary, NC). *P* < 0.05 was considered statistically significant.

## Results

In all cases, in the coronal images, a curvilinear low-intensity band that agreed with the signal intensity of silicone material [16,17], was seen at the location of the endplate was placed. In all cases, high-intensity areas with the same intensity as intraocular fluid were adjacent to the endplate, suggesting successful detection of the filtering bleb. Of interest, in all cases two layers of fluid accumulation were seen consistently in axial, coronal, or sagittal slices, suggesting that the bleb formed all around the endplate including beneath it.

In all 27 eyes, the preoperative IOP of 38.8±2.2 mmHg and medication score of 3.2±0.1 decreased to 13.6±0.7 mmHg and 1.7±0.2, respectively, at 6 months postoperatively (Table 1).

At this time period, the MRI-measured bleb volume, bleb height, and distances 1, 2, and 3 were calculated as 384.7±67.0 mm^3^, 3.5±0.3 mm, 6.0±0.1 mm, 9.4±0.2 mm, and 7.2±0.3 mm, respectively (Table 2).

**Table 2:** MRI-Measured Parameters.

Case	Bleb Volume (mm^3^)	Bleb Height (mm)	Distance 1 (mm)	Distance 2 (mm)	Distance 3 (mm)
1	329.0	3.9	5.5	9.4	4.8
2	61.3	3.1	6.9	9.0	7.5
3	272.2	4.2	6.1	9.1	6.1
4	8.4	2.1	5.4	8.0	6.3
5	45.4	2.2	5.9	8.5	6.8
6	51.6	2.2	5.4	9.1	6.0
7	129.5	2.6	6.0	9.4	6.0
8	391.3	5.2	6.6	10.0	9.0
9	646.0	4.1	4.3	6.8	6.0
10	472.8	3.8	7.1	10.8	8.3
11	1696.6	9.3	5.5	8.3	6.9
12	801.2	2.6	6.8	10.1	9.0
13	257.2	5.4	5.8	9.0	8.2
14	391.9	5	5.8	9.6	7.9
15	433.1	1.8	6.8	10.8	8.8
16	809.0	3.8	6.3	9.8	8.2
17	377.0	4.5	5.8	9.3	6.6
18	645.1	5.3	5.8	10.0	4.4
19	615.0	4.6	6.3	10.2	9.1
20	241.0	2.4	6.3	8.9	7.4
21	568.0	2.7	6.5	10.4	8.7
22	188.0	2.3	6.5	10.6	7.7
23	111.4	2.5	5.2	9.1	6.0
24	99.0	2.9	6.0	8.6	5.9
25	253.2	2.4	5.3	8.4	6.5
26	221.3	2.5	6.2	9.3	8.3
27	271.5	1.9	6.6	11.0	7.8
Mean	384.7	3.5	6.0	9.4	7.2
SEM	67.0	0.3	0.1	0.2	0.3

Distance 1 indicates the distance between the corneal limbus and the anterior edge of the shunt endplate.

Distance 2 indicates the distance between the corneal center and the anterior edge of the shunt endplate.

Distance 3 indicates the distance between the shunt endplate and the nearest orbital wall.

MRI, magnetic resonance imaging; SEM, standard error of mean.

**Fig 3:**
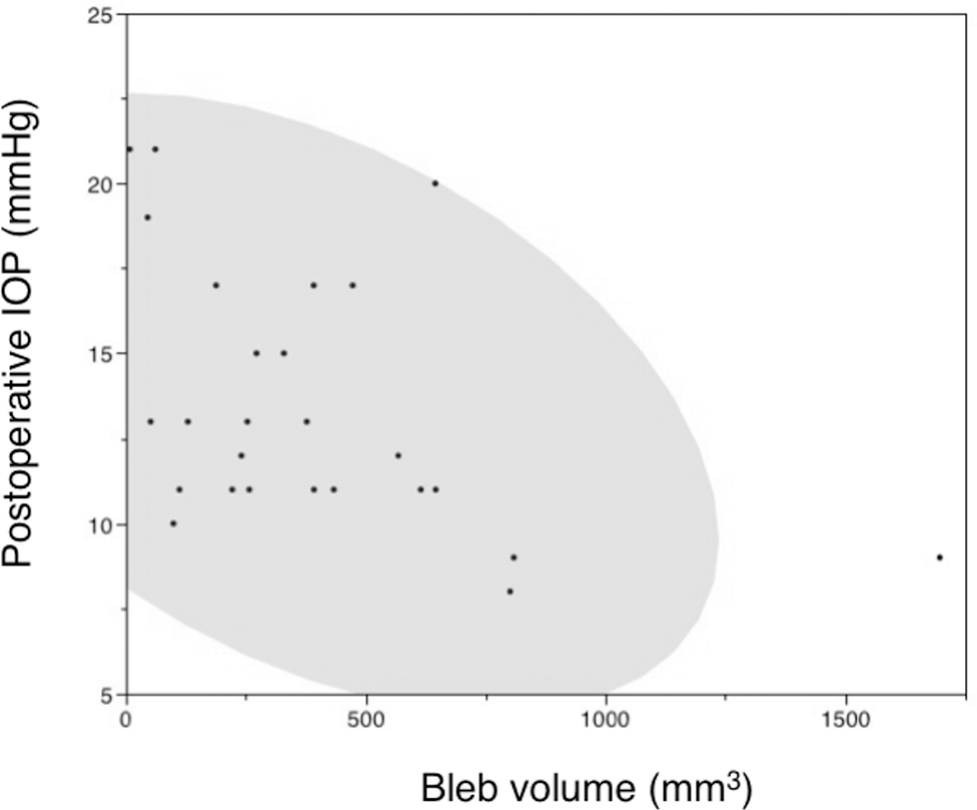
The correlations between the postoperative intraocular pressure (IOP) level and bleb volume. The oval area indicates a 95% bivariate normal ellipse.

By non-parametric correlation analyses, the bleb volume (r = -0.4510 and *P* = 0.0182) (Table 3, Fig 3) and bleb height (r = -0.3954 and *P* = 0.0412) (Table 3) were correlated negatively with the postoperative IOP; neither the preoperative IOP nor the reduction of the IOP was correlated with bleb volume. None of the comparison pairs between the distance parameters and IOP parameters were correlated with each other (Table 3).

**Table 3:** Correlations between IOP Levels and MRI-Measured Parameters.

	Preoperative IOP		Postoperative IOP		IOP Reduction	
	r	*P* Value	r	*P* Value	r	*P* Value
Bleb volume	-0.1873	0.3495	-0.4510	0.0182*	0.0021	0.9915
Bleb height	-0.0211	0.9169	-0.3954	0.0412*	0.0844	0.6754
Distance 1	0.2465	0.2151	0.0933	0.6433	0.1315	0.5132
Distance 2	0.0584	0.7723	0.1054	0.6008	0.0698	0.7294
Distance 3	0.1349	0.5025	-0.2511	0.2064	0.1656	0.4090

IOP, intraocular pressure; MRI, magnetic imaging. The r and P values indicate Spearman's rank correlation coefficient and calculated probability, respectively.

The asterisk (*) indicates *P*<0.05.

The clinical parameters and MRI-measured values were compared between the AGV and BGI groups (Table 4). The eyes implanted with the two different BGI models were analyzed as one group since the three eyes implanted with the BGI103-250 (n = 3) was too few to analyze independently. By non-parametric comparison analyses, the preoperative IOP was similar between groups (37.0±2.7 mmHg in the AGV group and 39.5±2.9 mmHg in the BGI group; *P* = 0.852), while the postoperative IOP level was lower and the IOP reduction was larger in the BGI group (12.2±0.7 mmHg and 27.4±2.9 mmHg, respectively) compared with the AGV group (16.7 ±1.2 mmHg and 20.3±3.1 mmHg, respectively), a difference in IOP that reached significance (*P* = 0.0026). The bleb volume and bleb height were larger in the BGI group (478.8±84.2 mm^3^ and 4.6±0.6 mm, respectively) than in the AGV group (161.1±52.3 mm^3^ and 3.2±0.4 mm, respectively), and the difference reached significance (*P* = 0.0093) for bleb volume (Table 4). No distance parameters differed between the AGV and BGI groups.

**Table 4:** Comparisons between Ahmed Glaucoma Valve (AGV) and Baerveldt Glaucoma Implant (BGI) groups.

	AGV Group	BGI Group	*P* Value
	(n = 8)	(n = 19)	
Age (years)	59.5±3.2	64.4±2.4	0.151
Sex (men/women)	7/1	12/7	0.365
Eye (right/left)	4/4	13/6	0.415
Glaucoma type (POAG/NVG and others)	3/5	7/12	1.000
Endplate position (ST/IT+IN)	1/7	4/15	1.000
Tube insertion (PP/AC)	7/1	16/3	1.000
Preoperative IOP (mmHg)	37.0±2.7	39.5±2.9	0.852
Postoperative IOP (mmHg)	16.7±1.2	12.2±0.7	0.0026**
IOP reduction (mmHg)	20.3±3.1	27.4±2.9	0.110
Preoperative medication score	3.0±0.3	3.2±0.1	0.678
Postoperative medication score	2.0±0.4	1.6±0.3	0.556
Bleb volume (mm^3^)	161.1±52.3	478.8±84.2	0.0093**
Bleb height (mm)	3.2±0.4	4.6±0.6	0.083
Distance 1	6.0±0.2	6.0±0.2	0.651
Distance 2	9.1±0.2	9.5±0.2	0.184
Distance 3	6.6±0.4	7.5±0.3	0.116

The *P* values are calculated between the AGV and BGI groups using the Mann-Whitney U test for continuous parameters and Fisher's exact probability test for categorical parameters. POAG, primary open-angle glaucoma; NVG, neovascular glaucoma; ST, superotemporal; IT, inferotemporal; IN, inferonasal; PP, pars plana tube insertion; AC, anterior chamber tube insertion; IOP, intraocular pressure. Data are expressed as mean ± SEM.

The double asterisk (**) indicates p<0.01.

## Discussion

Previous studies have used various imaging modalities to evaluate the postsurgical status of long-tube GDDs. High-frequency UBM was used to image the peritubular filtration in eyes implanted with the AGV [18], and anterior-segment OCT was used to visualize the AGV tube inserted into the anterior chamber [19] or pars plana [8]. Among the long-tube GDDs available, only the BGI is radiopaque because the endplate is made of barium-impregnated silicone; plain radiographs [20] and computed tomography [21] were used to assess the intraorbital presence of the BGI. A gadolinium-enhanced T1-weighted image was used to evaluate the status of the extraocular muscles around the BGI plate [22] and the fibrosis of the bleb wall around the AGV plate [23]. A T2-weighted image was used to visualize filtering blebs in eyes implanted with the AGV [24–26] or Molteno implant [25]. In the current study, MR image were obtained by 3D-FIESTA that enables acquiring high signal-to-noise ratio images with excellent spatial and temporal resolutions [27] to visualize and quantitate filtering bleb formation and endplate positioning in eyes implanted with the AGV and BGI.

We consistently observed two layers of intraorbital-periocular accumulation of ocular fluid beneath and above the endplate in eyes with both types of implants by the combined use of the head-array coils and the 3T-MRI device. During previous MRI observations of filtering blebs in eyes implanted with the AGV, it was noted that “the tube shunt could be identified within the fluid collection [24]” or that “a dark curvilinear band representing the silicone-made endplate of the implant is seen at the center of the cyst on both the T1- and T2-weighted images [26]” in two single case reports. Thus, the descriptions suggested the presence of two layers of fluid accumulation. Detorakis et al. assessed the filtering bleb by combined use of surface coils or head coils and 1.5T-MRI device in eight cases (4 AGV and 4 Molteno implant cases) [25]. Although they did not directly report the presence of two layers of fluid accumulation, their published images clearly depicted two layers of fluid accumulations in cases (cases 1 and 2 of their report) with the AGV and Molteno implants. Thus, our observation agreed with previous observations, and, therefore, suggested that the filtering bleb after implantation of long-tube GDDs is formed whole around the endplate including the space between the endplate and sclera; this is difficult to assess by modalities other than MRI. Collectively, detection of two layers by MRI can be a good indication of successful filtration, and, vice versa, the absence of two layers may indicate encapsulation of the filtering bleb as suggested previously [25].

We found a clear inverse association with the filtering bleb volume and the postsurgical IOP levels (Table 3, Fig 3), as previously suggested in eyes after trabeculectomy [28,29] and in eyes implanted with long-tube GDDs [25]. Our estimation of bleb volume (384.7 mm^3^) is much larger than the previous estimation in GDD-implanted eyes by Detorakis et al. (0.24 mm^3^ after AGV and 0.18 mm^3^ after Molteno implantations) [25]. Theoretically, considering the endplate area, e.g., the bleb volume 1 mm high over the 350 mm^2^ endplate is calculated to be 350 mm^3^, our estimation seems more reasonable than the previous report. The preoperative IOP levels or reductions in IOP were not correlated with the bleb volume (Table 3) or postoperative IOP (data not shown). Although the preoperative IOP is a major determinant of the postoperative IOP level in non-filtration surgery such as trabeculotomy [30,31], at least during the early postoperative phase for 6 months, GDD surgery reduced the IOP irrespective of the preoperative IOP levels, which is common among the filtration surgeries. We found no correlation between any of the three distance parameters of endplate position and postoperative IOP (Table 3). A previous report found a significant correlation between anterior positioning of the endplate and higher postoperative IOP [25]. In that report, two of eight cases had poor IOP control; thus, the absence of surgical failure in the current study may explain the discrepancy, but future clarification is needed.

We compared the MRI findings between two commonly used GDDs. We found a significantly larger bleb volume with the BGI (plate size, 250 or 350 mm^2^; bleb volume, 478.8 mm^3^) than with the AGV (plate size, 184 mm^2^; bleb volume, 161.1 mm^3^) (Table 4). Since the bleb height did not differ significantly between the groups, the difference in bleb volume is explained primarily by the difference in the expansion of the bleb along the endplate. In a previous study, the bleb volume was larger with the AGV (plate size, 184 mm^2^; n = 4) (bleb volume, 0.24 mm^3^) than with the single-plate Molteno implant (plate size, 129 mm^2^; n = 4) (bleb volume, 0.18 mm^3^), although the difference was not significant [25]. Based on the images obtained, we learned that the bleb merely extended far beyond the endplate; thus, most likely, the expansion of the bleb was afforded by the size of the endplate.

We found a significantly lower postoperative IOP level associated with the BGI (12.2 mmHg) than AGV group (16.7 mmHg) 6 months postoperatively (Table 4). This agrees with the early postoperative results in two major randomized studies that compared the AGV and BGI [32,33], although the results did not agree completely with previous retrospective studies [34,35]. A greater IOP reduction was reported in association with a device with a larger plate compared with single- (133 mm^2^)- and double (266 mm^2^)-plate Molteno implants [36]; however, in a comparison of the 350 mm^2^ and 500 mm^2^ BGIs, this plate size-mediated effect on the postoperative IOP was weaker after the intermediate term [37] and undetectable after the longer term [38]. Thus, the effect of the plate size on the IOP reduction can reach a plateau with the largest plate size. In experimental settings, both the flow resistance and pressure were higher in rabbit eyes implanted with the AGV compared with the BGI [39], and the flow resistance was correlated inversely with the plate size in a comparison between the 50, 100, and 200 mm^2^ BGIs, while the hydraulic conductivity of the bleb capsule was identical among the different plate sizes [40]. Collectively, we speculated that filtering blebs with a larger volume formed after implantation of the BGI compared with the AGV, which explains the lower postoperative IOP levels achieved with the BGI than the AGV in the range of plate sizes and postoperative periods that we studied.

The retrospective nature of this study may be related to a selection bias of the subjects; the sample size may not have been sufficiently large to fully estimate a possible correlation between the endplate positioning relative to the eyeball or orbital wall. The heterogeneous inclusion of various glaucoma types and surgical techniques also can be a study limitation. However, the incidence of each glaucoma types, placement of the endplate superiorly or inferiorly, and tube insertion into the anterior chamber or pars plana did not differ between the AGV and BGI groups among the current subjects (Table 4). The surgical outcomes were similar between the superior and inferior endplate placements after AGV implantation [41] and between the anterior chamber or pars plana tube insertions after AGV [42] and BGI [43] implantation. One surgeon performed all surgeries. We believe that our observations of an inverse correlation between the IOP and bleb volume and a difference in bleb volume between the devices are scientifically reasonable.

In conclusion, the presence of intraorbital-periocular accumulation of ocular fluid assessed on MR images obtained by 3D-FIESTA sequences affects the postoperative IOP level in eyes implanted with long-tube GDDs. Formation of a larger filtering bleb after BGI implantation compared with the AGI implantation explains the lower postoperative IOP levels achieved with the BGI. Recent studies have suggested that magic angle-enhanced MRI can be applied to detect the changes of ocular fibrous microstructures in sclera and cornea upon IOP elevation [44] and that gadolinium-enhanced MRI reveals aqueous humor dynamics on ocular hypertension [45]. In combination with these new strategies, we may have more insight on the mystery of IOP regulation after GDD implantation.
